# A fiber-optic system for dual-modality photoacoustic microscopy and confocal fluorescence microscopy using miniature components^☆^

**DOI:** 10.1016/j.pacs.2013.07.001

**Published:** 2013-08-07

**Authors:** Sung-Liang Chen, Zhixing Xie, L. Jay Guo, Xueding Wang

**Affiliations:** a Department of Radiology, University of Michigan, Ann Arbor, MI 48109, United States; b Department of Electrical Engineering and Computer Science, University of Michigan, Ann Arbor, MI 48109, United States

**Keywords:** PAM, photoacoustic microscopy, CFM, confocal fluorescence microscopy, MEMS, microelectromechanical systems, 2D, two-dimensional, 1D, one-dimensional, FWHM, full width at half maximum, MAP, maximum amplitude projection, Photoacoustic microscopy, Confocal fluorescence microscopy, Microring resonator, Microelectromechanical systems, Endoscopy, Bladder cancer

## Abstract

Imaging of the cells and microvasculature simultaneously is beneficial to the study of tumor angiogenesis and microenvironments. We designed and built a fiber-optic based photoacoustic microscopy (PAM) and confocal fluorescence microscopy (CFM) dual-modality imaging system. To explore the feasibility of this all-optical device for future endoscopic applications, a microelectromechanical systems (MEMS) scanner, a miniature objective lens, and a small size optical microring resonator as an acoustic detector were employed trying to meet the requirements of miniaturization. Both the lateral resolutions of PAM and CFM were quantified to be 8.8 μm. Axial resolutions of PAM and CFM were experimentally measured to be 19 μm and 53 μm, respectively. The experiments on *ex vivo* animal bladder tissues demonstrate the good performance of this system in imaging not only microvasculature but also cellular structure, suggesting that this novel imaging technique holds potential for improved diagnosis and guided treatment of bladder cancer.

## 1 Introduction

Bladder cancer is the fifth leading new cancer diagnosis in the United States and is the fourth among men [1]. Despite the quick advances in both diagnosis and therapy in the past decades, bladder cancer remains an important public health problem. One of the reasons is the lack of powerful screening and imaging technologies. The study of tumor angiogenesis and microenvironments plays an important role in cancer diagnosis. Photoacoustic microscopy (PAM) is an emerging technique for microscopic imaging of optical absorption contrast and has been demonstrated as a useful tool in mapping angiogenic microvasculature in biological tissues *in vivo* [2]. On the other hand, fluorescence imaging technologies have been increasingly applied to assessment of tissue pathology, especially in imaging specific anatomical structures with autofluorescence or labeled with fluorescent dyes [3], [4]. Successful applications of imaging by utilizing either photoacoustic or fluorescence contrast to cancer detection and characterization have been demonstrated [5], [6], [7].

PAM and confocal microscopy, enabling high-resolution imaging of microvascular and cellular structures within a thin optical section respectively, have been widely used in many other areas [8], [9]. Both techniques can be achieved through optical focusing and provide *in vivo* imaging with micrometer resolution to depth of several hundred micrometers [10], [11]. Numerous *in vivo* applications were in the organs which is relatively accessible, such as eye [12], [13] and skin [14], [15]. Development of compact scanning heads has facilitated the endoscopic applications of PAM and confocal microscopy [16], [17], [18]. Imaging in the endoscopic manner, similar to the procedure of conventional white light cystoscope, is probably the best method to achieve *in vivo* evaluation of non-muscle invasive bladder tumors.

Dual modality systems combining PAM and confocal fluorescence microscopy (CFM) have been demonstrated based on bulky components [19], [20]. Despite the rapid advances of endoscopic PAM and CFM in the past years, building a miniaturized scanning probe capable of acquiring the two optical contrasts simultaneously both with high spatial resolution has not yet been realized. The two complementary contrast mechanisms associated with PAM and CFM are sensitive to microvasculature and individual cells respectively. Presenting more comprehensive diagnostic information, such a dual-modality endomicroscopic system, if achievable, could show promise for improved detection and guided treatment of non-muscle invasive bladder tumors. Moreover, imaging microvasculature and individual cells at the same time may also enable *in vivo* investigation of the interaction of cancer cells with ambient microenvironments, and help better understand the mechanism of disease onset, progression and responses to therapy.

To achieve optical resolution PAM and CFM through an integrated system, the optical beam needs to be focused and scanned across the sample. Besides, the focusing and scanning should better be implemented at the distal end of a probe, which is usually a few millimeters in diameter in order to fit the requirements for endoscopy. In confocal microscopy, the use of piezoelectric actuator to physically deflect the tip of an optical fiber has been demonstrated [21]. This method for mechanical scanning at the distal of a probe, however, imposes difficulties on miniaturization. In another method, the optical scan was achieved through a fiber-optic bundle binding thousands of individual fibers [22], circumventing mechanical scanning at the tip of a probe. This method, although easy to implement, is suffered from the inherent pixilation artifact due to the finite spacing between adjacent fibers and the limited field of view restricted by the size of the fiber bundle.

The recent advancement in miniaturized scanning mirrors based on microelectromechanical systems (MEMS) technology has enabled the feasibility of fabricating compact fiber-optic-based endomicroscopic probes [23], [24], [25], [26]. In our previous work, we have successfully built an all-optical MEMS-based PAM system using miniature components and achieved imaging of microvasculatures inside a canine bladder wall [27]. In this study, we further incorporate CFM imaging modality into this system without imposing any burden on size at the probing end, which serves as a prototype for future development of an endoscopic probe. Besides, a higher numerical aperture objective lens with a smaller size is used, which further improves the lateral resolution. Through the experiment on bladder specimens, we have validated the performance of this system in imaging microvasculature and single cells based on the optical absorption and fluorescent contrasts respectively. The excitation light, the fluorescent light, and the optical signal from the microring detector are all delivered through optical fibers. Such a fiber-based all-optical design will benefit future development on a miniaturized endoscopic probe for clinical applications considering the small size of fibers. Besides, the good flexibility of fibers is also beneficial because bending of a probe in human inherent passages such as the urethra or the esophagus is inevitable during diagnosis or treatment.

## 2 Methods

**Fig. 1:**
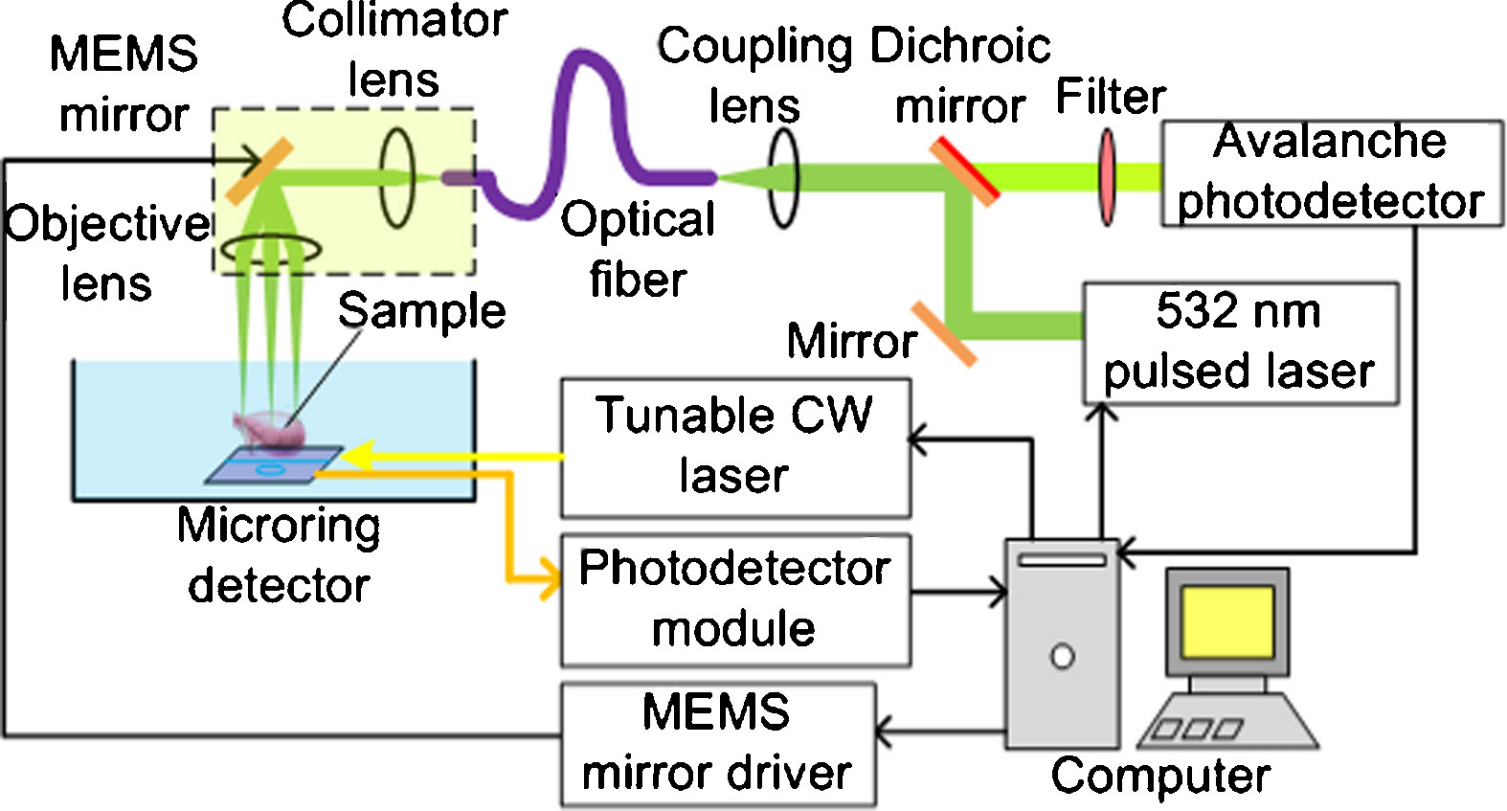
Schematic diagram of the fiber-optic based PAM and CFM dual-modality imaging system. The light input and output for the microring detector are through optical fibers. The dashed box indicates the probe part of the prototype system.

A schematic diagram of the fiber-optic based PAM and CFM dual-modality imaging system is shown in Fig. 1, where the dashed box indicates the probe part of the prototype system. The irradiation light source is a diode-pumped solid-state Nd:YAG laser (SPOT-10-200-532, Elforlight Ltd., UK) with a wavelength of 532 nm and pulse duration of 2 ns. The collimated beam from the laser head is reflected by a mirror and is then reflected by a dichroic mirror (DMLP567, Thorlabs, Newton, NJ). After that, the light is coupled into a fiber (core diameter: 11 μm, SM2000, Thorlabs) using a fiber port coupler (PAF-X-5-A, Thorlabs). The larger core size than that of a single mode fiber for 532 nm light is chosen for better coupling, allowing sufficient light energy for photoacoustic generation. At the other end of the fiber, light emerges and is then collimated to a diameter of ∼2 mm (collimator: lens diameter ∼6 mm, CFC-8X-A, Thorlabs) before delivering to a MEMS mirror (mirror size: 2.5 mm × 2.0 mm, outer diameter: 9.2 mm, TM-2520, Sercalo Microtechnology Ltd., Switzerland). A miniature aspheric lens (outer diameter: 4.7 mm; focal length: 6.16 mm, 48147, Edmund Optics, Barrington, NJ) is used as an objective lens.

Two-dimensional (2D) raster scan of laser beam is performed by the MEMS mirror and two pairs of electrodes for electrostatic actuators. The mirror tilt angle is controlled by a driver board, comprising a digital-to-analog converter to deliver voltages to set the designated angles. By coding different voltages of the two pairs of electrodes, the two axes can be controlled to realize 2D scan. The scan is designed to perform fast in one axis and slow in the other. The maximum angular deflections are ±6° and ±9°, resulting in a large field-of-view of 1.29 mm × 1.95 mm considering the 6.16 mm focal length of the objective lens. More details of the MEMS mirror used in this work can be found in our previous work [27]. Due to the 50 Hz limit of the MEMS driver board, a 2D raster scan of 256 × 256 steps takes almost half an hour, which also includes the data transfer time. However, as we know, a MEMS mirror with step response setting times <100 μs is commercially available, which can drastically improve the imaging speed. Considering the same 2D scan over 256 × 256 points, the speed improvement gained by using the state-of-the-art MEMS mirror can be over two orders of magnitude, resulting in a 7-second scanning time which should meet the requirement for *in vivo* imaging.

In PAM imaging, photoacoustic signal is detected by an optical microring resonator, serving as a sensitive and broadband (bandwidth up to ∼100 MHz) ultrasonic detector [28]. The chip size of microring resonators can be made as small as 3.5 mm [29]. The microring detector with a ring diameter of 60 μm is placed under the sample, working on a transmission mode. The design and working principles of the microring resonator as an ultrasonic sensor have been introduced in our previous work [28], [30]. Briefly, acoustic pressure modulates the resonance condition, leading to a shift of the resonance wavelength. When the microring is probed at a fixed wavelength with a high slope in the transmission spectrum, the input ultrasound wave translates into the output optical intensity, which is then recorded by a high-speed photodetector. Therefore, high quality factor enables high-sensitivity detection. The acoustic field of view (or the angular response) of the microring detector depends on the ring size and the acoustic bandwidth of interest. For the current device with a diameter of 60 μm, the receiving angle is 40° for 20 MHz bandwidth at −6 dB [28]. During laser scan, the microring detector is kept stationary. A photoreceiver module (1801-FC-AC, New Focus) is used to detect pressure-modulated optical signal through the microring. The received signal is then recorded by a digitizer (CS22G8, DynamicSignals LLC, Lockport, IL) at a sampling rate of 1 GHz for 2 μs. No signal averaging is performed.

The two imaging modalities share the same scanning optical path and laser source. In CFM imaging, the back-traveling fluorescent light returning from the sample is collected by the objective lens. After coming out from the fiber, the light passes through the dichroic mirror and a longpass filter (FEL0550, Thorlabs) to block the light at excitation wavelength. The fluorescent light at wavelength above 550 nm transmits and is detected by an avalanche photodetector module (APD110A, Thorlabs). The amplified electrical output from the avalanche photodetector module is also digitized by the same digitizer used in PAM imaging. In this design, we ensure confocal detection by using the fiber with a small core size which plays the same role as the pinhole in a conventional confocal microscope [31].

## 3 Results

**Fig. 2:**
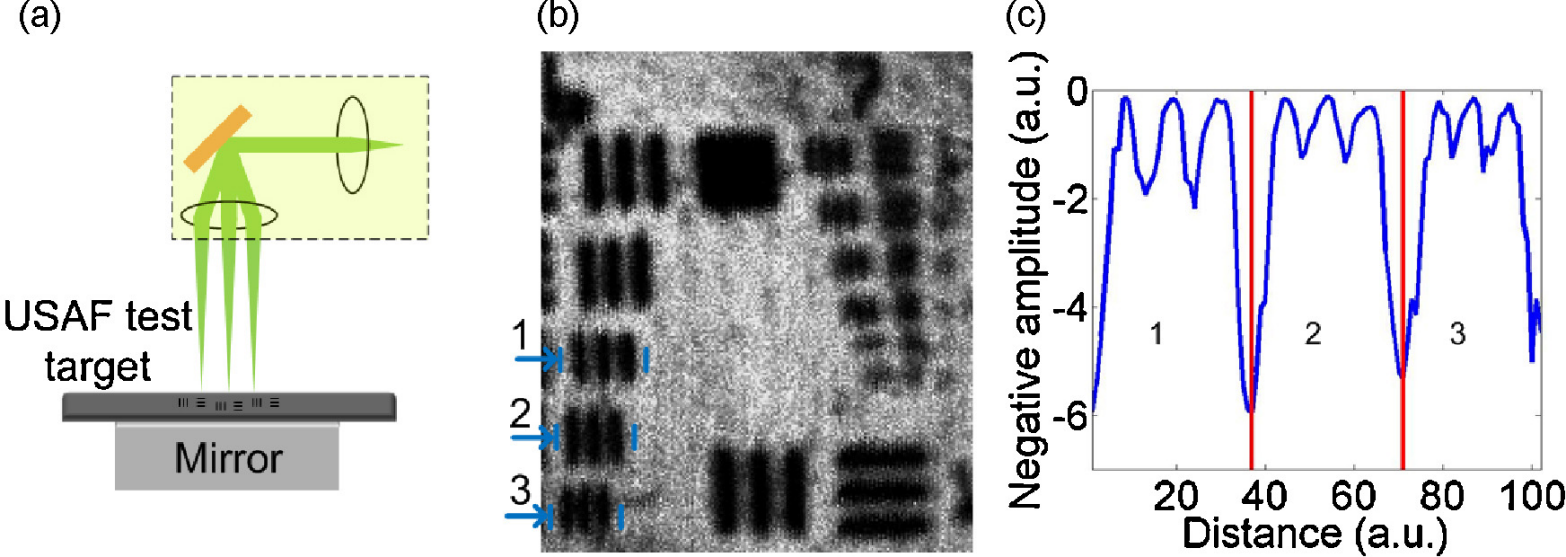
(a) Schematic diagram of a USAF resolution test target placed above a mirror and scanned with the imaging system. (b) The confocal microscopic image of the USAF test target. (c) 1D amplitude profile plotted along the lines marked in (b).

We first calibrated the lateral resolution of this system in the CFM imaging mode. The lateral resolution was quantified by imaging a USAF 1951 resolution test target (T-20-P-TM, Applied Image, Rochester, New York). The test target was placed above a mirror and scanned with the imaging system, as shown in Fig. 2(a). The wavelength of 532 nm, which will be used for fluorescence excitation, was used. The confocal microscopic image of the test target is shown in Fig. 2(b). Fig. 2(c) shows the one-dimensional (1D) amplitude profile measured along the lines marked in Fig. 2(b). Spatial averaging over 12 pixels was applied to improve the contrast-to-noise ratio. Determined by this resolution test target (group 6, element 6), the lateral resolution in the CFM mode was 8.8 μm.

The definition of 1 airy unit is 1.22 × λ/NA, where λ is the optical wavelength and NA is the numerical aperture of an objective lens. When the pinhole size of a confocal microscopy system is larger than 1 airy unit, which is 4.0 μm [=1.22 × (0.532 μm)/(2 mm/(2 × 6.16 mm))] in our case, the lateral resolution of confocal microscopy will be the same as that of conventional microscopy if the same optical components are used. Considering an 11 μm pinhole size, the lateral resolutions of our system in conventional and confocal microscopy modes are the same. The lateral resolution of optical-resolution PAM is determined by optical focusing [8], the same as conventional optical microscopy. Thus, the lateral resolution of PAM is also 8.8 μm. Compared with our previous work [27], the current system is improved about twice in lateral resolution by using the objective lens with higher numerical aperture.

**Fig. 3:**
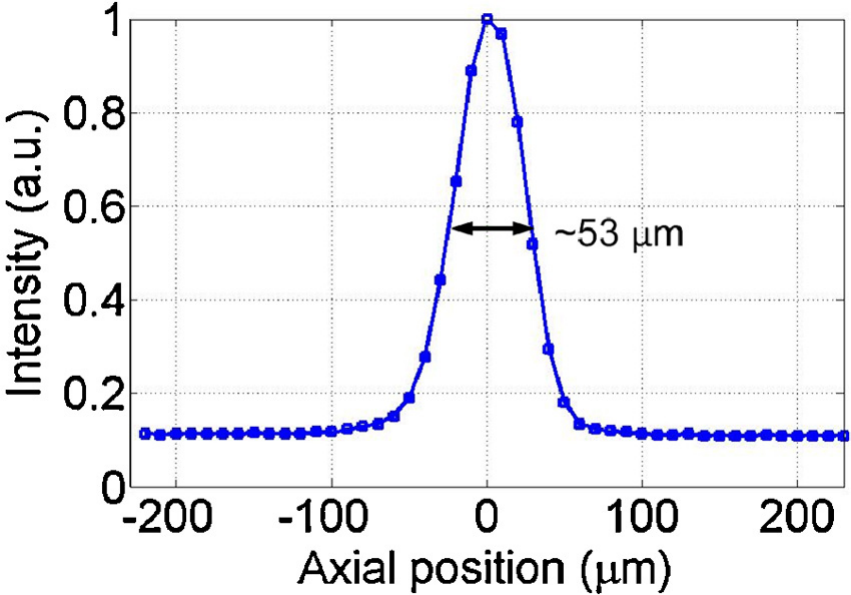
The measurement of axial resolution for CFM imaging modality, showing the FWHM of 53 μm. This intensity profile was measured when a mirror was translated axially through the focus.

To demonstrate the optical sectioning ability of CFM, the axial resolution of CFM was calibrated. Similarly, the system was working on confocal reflection microscopy mode. A flat mirror was moved along axial direction and the corresponding response is shown in Fig. 3. Determined by its full width at half maximum (FWHM), the axial resolution is 53 μm. The low axial resolution of CFM is in part due to the large core size of the fiber used, which is necessary for photoacoustic excitation, as mentioned above. In the future, we aim to use a fiber fusion splicing technique to make a fiber with large core size at one end and small core size at the other to accommodate both high coupling efficiency and small pinhole size which are essential for high-quality PAM and CFM at the same time. Another way to improve the axial resolution of CFM is to involve custom-designed lenses with high numerical aperture in a small size, as demonstrated in the literature [32].

**Fig. 4:**
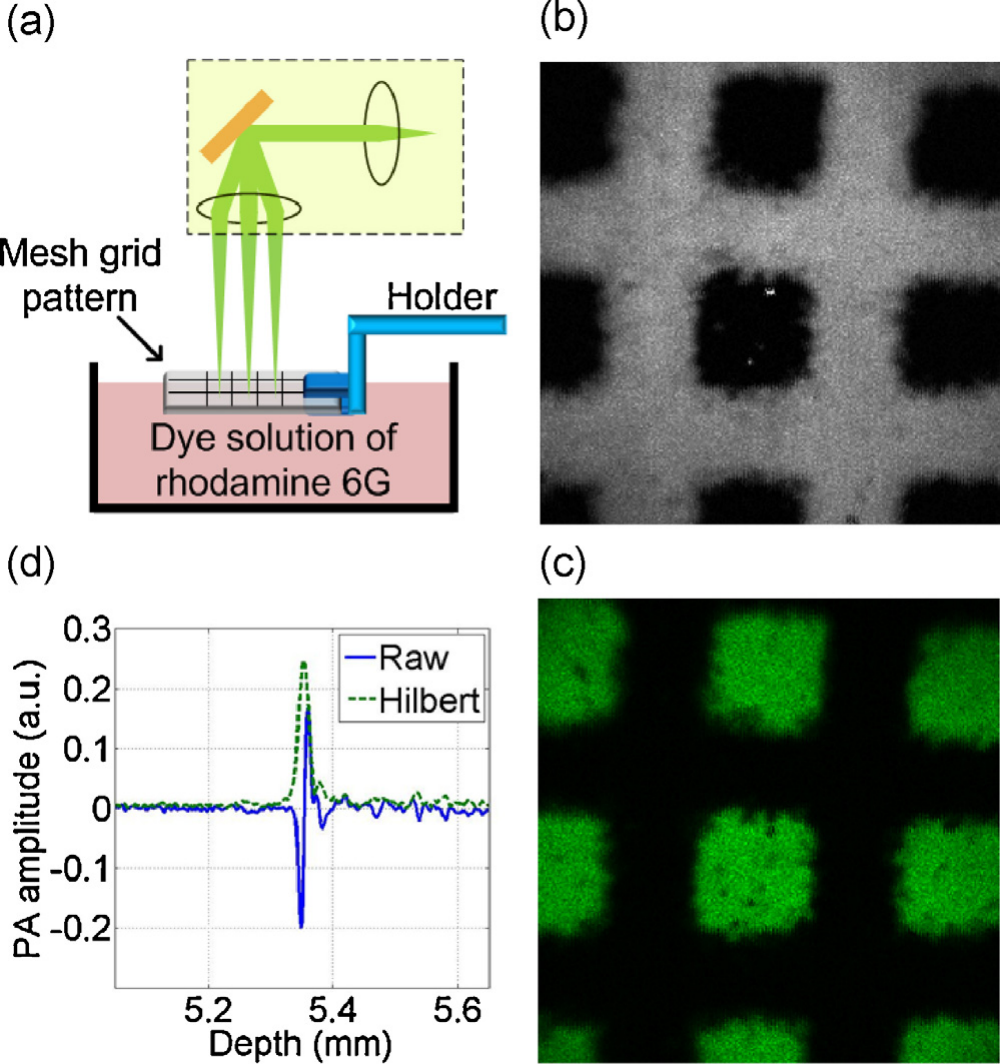
(a) Schematic diagram of the phantom used in this experiment. The phantom consists of a mesh grid pattern printed on a transparency which is placed above a diluted dye solution of rhodamine 6G. The transparency is fixed by a holder. (b) PAM MAP image and (c) CFM image of a black mesh grid printed on a transparency with a diluted dye solution of rhodamine 6G underneath. (d) A typical A-line photoacoustic signal from the phantom and its amplitude after the Hilbert transform, showing the FWHM of 19 μm.

To demonstrate the dual imaging ability of the system, we first imaged a phantom which consists of a black mesh grid printed on a transparency placed above a diluted dye solution of rhodamine 6G, as shown in Fig. 4(a). Rhodamine 6G has its absorption maximum at 530 nm with peak emission of 566 nm. Such a phantom provides both of the two contrasts, generating photoacoustic signal on the black mesh grid and fluorescent signal from the dye solution. The scanning area was 0.9 mm × 0.9 mm. The 2D PAM image was obtained by the maximum amplitude projection (MAP) in the scanning plane. Fig. 4(b) and (c) shows the PAM and CFM imaging results of the phantom, respectively. Since the two images were acquired through a single device, they are co-registered naturally. These two images showing the complementary optical information in the phantom demonstrate the system capability of conducting PAM and CFM simultaneously. Fig. 4(d) presents a typical A-line photoacoustic signal from the phantom. By studying its Hilbert transform and using the criteria of the FWHM, the axial resolution of the system in the PAM mode is determined to be better than 19 μm. Higher axial resolution has been achieved using the same microring detector when the sound propagation distance was smaller [33]. In contrast, the fluorescent signal is a monopolar and its peak amplitude was used.

**Fig. 5:**
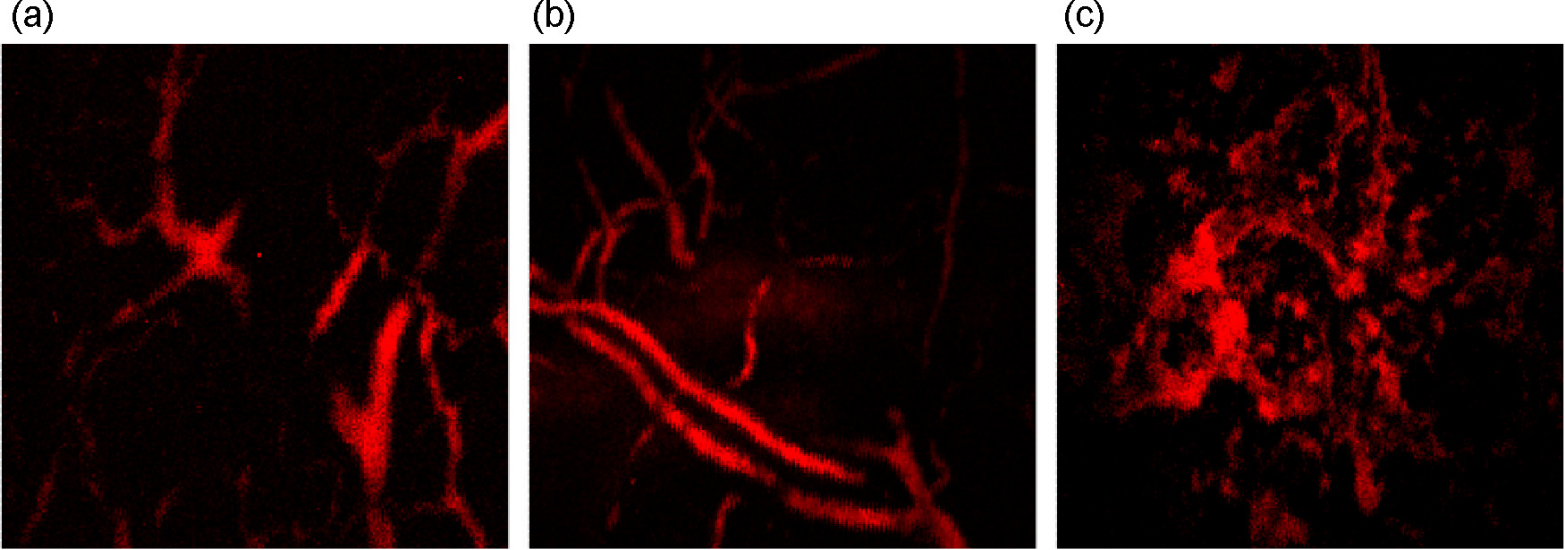
PAM MAP images of the microvasculature in a canine (a), a rat (b), and a pig (c) bladders. The scanning areas were all 0.9 mm × 0.9 mm.

For *ex vivo* tissue imaging, fresh animal bladders, including those from dogs, rats and pigs, were obtained under animal protocols approved by the UCUCA of the University of Michigan. This study on bladder models helps exploring the potential of adapting the dual-modality imaging system to future clinical management of bladder cancer. Fig. 5(a)–(c) presents example PAM images of the microvasculature in bladder tissues, each from different animal models. Based on the endogenous optical absorption contrast, the microvasculatures with different sizes of vessels in the bladder specimens were clearly imaged. Compared with the results from the canine and the rat bladders, more blood clots can be seen in the pig bladder, as shown in Fig. 5(c), because the pig bladder specimens from the slaughter house were not as fresh.

**Fig. 6:**
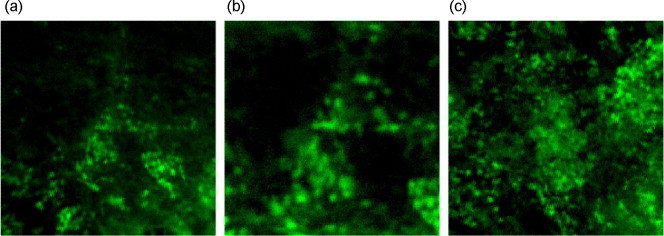
(a) CFM image of a canine bladder with a scanning area of 0.6 mm × 0.6 mm. (b) The enlarged CFM image of the central part of (a) with a scanning area of 0.3 mm × 0.3 mm. (c) CFM image of another canine bladder specimen with a scanning area of 0.6 mm × 0.6 mm.

Other than the PAM imaging, we also acquired CFM images from the canine bladder specimens, as shown in Fig. 6. We diluted a stock solution of rhodamine 6G in ethanol to a concentration of 10 mM, and then further diluted in phosphate buffered saline to a concentration of 100 μM. The fresh canine bladder was immersed in the diluted solution for 30 min, and then was rinsed with saline before imaging. As shown in Fig. 6, the individual cells with sizes down to ∼10 μm can be clearly discerned. The cell sizes recognized in CFM images are in good agreement with the findings from the literatures [34], [35], which is another proof of the cellular structure visualized in Fig. 6.

## 4 Discussion

Although this work has demonstrated the possibility to achieve PAM and CFM dual-modality endomicroscopy by using the all-optical design, further developments are needed before this technique is optimized for clinical applications. First, a MEMS mirror with an element size less than 3 mm and a faster scanning speed is crucial to satisfy the medical standard for cystoscopic catheters. As we know, MEMS mirrors with chip sizes of ∼3 mm is currently available and has been used in endoscopic imaging probes [25], [26]. Second, microring resonators could be made on an optically transparent substrate. In the current system using a microring detector on a nontransparent substrate, PAM is operated either in a transmission mode or with the microring placed at the side and next to the scanning laser beam. None of these two geometries are optimized. With the microring on an optically transparent substrate, the scanning laser beam can then go through the microring without being interfered. In this way, the PAM can be performed in a reflection mode with the microring detector contacting the tissue directly, avoiding unnecessary signal attenuation due to the long acoustic path. The feasibility of building an optically transparent microring detector for reflection-mode photoacoustic imaging has been demonstrated in another work [36]. Third, although the improvement in lateral resolution can be achieved by increasing the numerical aperture of the objective lens, the accompanied decrease in field of view has to be considered. Therefore, the balance between the lateral resolution and the field of view should be considered and adjusted based on different applications.

Our system is capable of simultaneously accessing the two different optical contrasts in biological tissues with PAM and CFM respectively. Such characteristic is crucial to achieve real-time inspection of the transient interactions between individual cells and ambient microenvironments. Although unlike CFM, the signal of PAM is from non-radiative relaxation, both CFM and PAM are generated by the optical absorption. Therefore, the events of CFM and PAM happen at the same time and the same location in the tissue and, therefore, could be synchronized exactly. For CFM of bladder tissues, further investigations on which layer (*e.g.*, superficial, intermediate, or basal) was stained assisted by histology sections would be of clinical value. The cell dimensions at different layers are important information for doctors to distinguish normal and malignant bladder tissues [35].

Our prototype system at the current stage is not able to perform transurethral imaging of bladder *in vivo* due to the technical issues discussed above. For demonstration of co-registered microvasculatures and cellular structures in *ex vivo* tissues, we suffered from undesired losing of blood from the tissues when immersing them in dye solution. The longer the *ex vivo* tissues are immersed in dye solution the more the blood will be washed out of the vessels, which will directly affect the performance of PAM. On the other hand, insufficient staining of cells results in poor quality of CFM images of cellular structures. Therefore, co-registered CFM and PAM images from *ex vivo* tissues are not presented. However, we do not expect same problem for future clinical or preclinical imaging *in vivo*, because treatment with fluorescent dye will not lead to losing of blood from live tissues [37].

## 5 Conclusions

Although both photoacoustic imaging and fluorescence imaging have been rigorously studied in the past years for their potential clinical applications including those to bladder cancer detection, integrating the two modalities into a single miniaturized device facilitating endoscopic imaging is still challenging and still has not been realized. To pave the road toward a multi-modality endomicroscope for better diagnosis of bladder cancer, in this work we tested the feasibility to achieve PAM and CFM dual-modality imaging by using a fiber-optic system and miniature components, including a MEMS-based scanning mirror, a miniature objective lens, and an optical microring ultrasonic detector. In the experiments on animal bladder models, promising results in mapping both microvasculature and individual cells in biological samples have been achieved, suggesting that this all-optical dual-modality imaging system, although needs further development, holds promise for endoscopic applications. Future studies will focus on further miniaturization and housing to realize a clinically usable endomicroscopic probe, as well as preclinical testing of the device on animal models.

## Conflict of interest

The authors declare that there are no conflicts of interest.
